# The Regulatory Landscape of Biobanks In Europe: From Accreditation to Intellectual Property

**DOI:** 10.2174/0113892029313697240729091922

**Published:** 2024-07-30

**Authors:** Antonella Corradi, Giuseppina Bonizzi, Elham Sajjadi, Francesca Pavan, Marzia Fumagalli, Luigi Orlando Molendini, Massimo Monturano, Cristina Cassi, Camilla Rosella Musico, Luca Leoni, Chiara Frascarelli, Oriana Pala, Elena Guerini Rocco, Adriana Albini, Roberto Orecchia, Nicola Fusco

**Affiliations:** 1 General Directorate for Libraries and Copyright, Italian Ministry of Culture, Rome, Italy;; 2 Biobank for Translational and Digital Medicine, European Institute of Oncology IRCCS, Milan, Italy;; 3 Division of Pathology, European Institute of Oncology IRCCS, Milan, Italy;; 4 Department of Oncology and Hemato-Oncology, University of Milan, Italy;; 5 Quality, Accreditation and Clinical Risk Service, European Institute of Oncology IRCCS, Milan, Italy;; 6 Technology Transfer Office, European Institute of Oncology IRCCS, Milan, Italy;; 7 Value Medicine, Clinical Risk and Privacy Area, European Institute of Oncology, IRCCS, Milan, Italy;; 8 Scientific Directorate, European Institute of Oncology IRCCS, Milan, Italy

**Keywords:** Biobanks, translational research, clinical studies, transparency and consent, biological materials, biomarkers

## Abstract

Biobanks are necessary resources for the storage and management of human biological materials, such as biofluids, tissues, cells, or nucleotides. They play a significant role in the development of new treatments and the advancement of basic and translational research, especially in the field of biomarkers discovery and validation. The regulatory landscape for biobanks, which is necessary to safeguard both privacy and scientific discoveries, exhibits significant heterogeneity across different countries and regions. This article outlines the standards that modern biobanks should fulfill in the European Union (EU), including general, structural, resource, process, and quality requirements. Special attention is given to the importance of transparency and donor consent following the General Data Protection Regulation (GDPR) and the ISO 20387:2018, the international standard specifies general requirements for biobanks. A dedicated section covers the preparation of donor information materials, emphasizing consent for research involvement and personal data processing. The delicate balance between donors' privacy rights and scientific research promotion is also discussed, with a focus on the patenting and economic use of biological material-derived inventions and data. Considering these factors, it would be warranted to refine legal frameworks and foster interdisciplinary collaboration to ethically and responsibly expand biobanking.

## 1 INTRODUCTION

### 1.1 Balancing between Timeliness in Research and Data Protection

Biobanks play a vital role in the global life science landscape as resources for the collection and preservation of biological material, usually human and associated data [1]. They serve various purposes, including, biodiversity studies, research endeavors, and technological development [2]. Human biobanks in the European Union (EU) are usually managed by or strictly interconnected with healthcare institutions, as well as research organizations [3]. Several types of biobanks are present, *e.g*. comprehensive biobanks, pathology archives, biobanks for security and crime prevention, organ transplantation, and stem cell facilities [4, 5]. The term “biobanking” emerged in the 1990s to delineate the practices associated with the archival and conservation of biospecimens, including organs, tissues, biofluids, and cells [6, 7]. Modern biobanks strive to adhere to internationally recognized guidelines, aiming to advance life science for the benefit of donors, their families, and the overall society [8-10]. By compiling biographical, genealogical, and clinical data, biobanks assemble a wealth of information encompassing not only human clinic-biological data but also its functional products [11, 12].

To uphold operational efficiency and timeliness and safeguard donors' personal data, biobanks should adhere meticulously to designated regulatory requirements and stringent quality standards [13, 14]. Embracing a formal recognition of their competence (in a single word: accreditation) becomes paramount for biobanks, fostering uniformity and consistency in their endeavors [15]. The International Organization for Standardization (ISO) develops consensus-driven global standards that facilitate innovation and address global challenges [15-17]. For biobanks, the ISO 20387:2018 defines the basic standards for competency, impartiality, and consistency [18]. According to ISO 20387:2018, biobanks can obtain accreditation for their operations [19, 20]. On the other hand, effective research often requires the association of biological material with sensitive donor data such as age, lifestyle, medical history, environmental conditions, and clinicopathologic and genetic information [21, 22]. While this information is indispensable for research purposes, it also poses risks to the privacy of the individuals involved [20, 23]. The EU General Data Protection Regulation (GDPR) in 2016 and its implementation in May 2018 were eagerly awaited within the biobank community [6]. The focus of this regulation in EU law on data protection and privacy has reshaped the landscape of biobank regulations.

However, discrepancies in these regulations might pose significant obstacles to effective and timely research.

This article aims to highlight the role of biobanks in global life sciences, emphasizing their significance in collecting, preserving, and managing human biological material and data. We discuss in detail the strategies to pursue biobank operational efficiency and accreditation, particularly in the EU, for impactful and timely research in accordance with international standards and privacy regulations. However, in the case of 20387, the harmony between its objectives and privacy protection suggests a deeply ingrained commitment to safeguarding the confidentiality and security of donors' information. This seamless integration reflects a strong dedication to upholding privacy while pursuing the organization's mission and objectives. It serves as a testament to their clarity and coherence in addressing both privacy concerns and broader organizational goals.

## 2 BIOBANK LEGAL FRAMEWORK

### 2.1 Data Protection Regulation in Europe and Beyond

Biobanks function under several multi-level regulatory frameworks, which encompass local and central regulations guaranteeing the safeguarding of research participants and guidelines for the utilization of pertinent material [24-27]. In Europe, the GDPR sets strict standards for the processing and protection of personal data [28]. Ethics reviews of research procedures assist biobanks in aligning with GDPR, but they may not fully address complex legal aspects, particularly those related to local and ultra-specialistic matters [17]. The GDPR applies to all EU members and has significant implications for biobanks within the various jurisdictions [29]. It emphasizes the importance of obtaining consent from donors, ensuring data pseudonymization where appropriate, implementing robust safety measures, and establishing clear mechanisms for data access and sharing. Beyond the EU, other countries have also developed legislation and regulations concerning biobanks. For instance, the United States Health Insurance Portability and Accountability Act (HIPAA) governs the privacy and security of health information [30]. Besides this federal law, various state laws and regulations address specific aspects of biobanking, research ethics, and data protection. Furthermore, international organizations such as the World Health Organization (WHO), the International Society for Biological and Environmental Repositories (ISBER), and the International Council for Harmonization (ICH) of Technical Requirements for Pharmaceuticals for Human Use have issued guidelines and best practices to guide the establishment and operation of biobanks on a global scale. Collectively, although the legal framework for biobanks differs across countries, it is clear that international regulations and guidelines are in place to guarantee the appropriate operation, privacy safeguards, and ethical standards of biobanks. A key challenge remains the absence of dedicated biobanks compling with avaible legislation in most regions of the world.

### 2.2 General and Structural Requirements

Biobanks should operate within an established organizational structure [22, 31]. The structure of the biobank may vary depending on its nature and resources and should be available to interested parties, possibly on the institution's website [32]. For each project, the organizational structure helps to identify the departments involved and their responsibilities. In addition, the biobank should have a clear organizational chart detailing the relationships between units, including the unit that oversees the quality management system (QMS). It is recommended that all relevant roles and positions should be included in the organizational char. That subordination to advisory bodies is outlined either in procedures or in the organization’s by-laws [33]. Depending on their size and mission, biobanks require different types of personnel [34]. Well-trained staff, working closely with the General Manager and the Quality Manager, should form the core of the biobank [34]. Quality standards such as ISO 20387:2018 provide specific guidance on staff qualifications [34]. To oversee compliance with data processing procedures, it is highly advisable to appoint a data protection officer (DPO) [12, 14, 29, 30, 35]. The DPO is also required to keep a register of all the processing operations carried out by the institution that involve the processing of personal data. The Register should be accessible to any interested party and contain clear information on the purposes and conditions of the processing operations [6].

### 2.3 Process Conditions: Compliance, Training, and Storage Management

Resource requirements encompass personnel, dedicated facilities/areas, equipment, reagents, information systems, and support services necessary for biobanking operations [2, 36]. Personnel involved in biobanking activities should undergo continuous training and receive operational instructions specific to their roles [20, 30]. Training plans should be based on regular evaluation of individual skills. Dedicated facilities/areas that meet defined quality control (QC) criteria, including fitness for the intended purpose, biosafety, and biosecurity, should be established and maintained [30]. External suppliers involved in the processing of biological material and/or associated data should be assessed and contracted as data processors under the GDPR or its equivalent in non-European settings. Biobank laboratory workflow encompasses various steps, including collection, accession, acquisition, identification, storage, long-term preservation, QC, transport, and disposal [37]. Archiving has an active role in biobank laboratory workflows, extending beyond the simple storage of biomaterials [38]. Archives with increasing numbers of tissue slides, formalin-fixed paraffin-embedded (FFPE) blocks, frozen tissues, and biofluid aliquots present an increasing challenge to the management of space [39]. Maintaining controlled temperature and humidity while ensuring security and traceability is essential for biobank archiving [39, 40]. Addressing these challenges requires a critical evaluation of the return on investment in modernizing archiving processes, particularly with the emergence of digital pathology and automation [40]. The advent of digital tools and innovations offers promising solutions that enable efficient sample preservation and tracking, thereby improving laboratory effectiveness, efficiency and ultimately patient health outcomes [38, 40]. To overcome the existing archiving barriers and improve archiving strategies, this procedure requires a comprehensive healthcare policy [1, 39, 41]. In this context, it is crucial to involve a data protection expert to ensure compliance with applicable regulations and to create a data flow map.

### 2.4 Quality Management System (QMS) and ISO 20387:2018

The quality standards function as crucial tools for the biobank stakeholders (*e.g*., scientists, individual participants, organizations, and companies). The ISO 20387:2018 delineates biobanks’ requirements grouped into five principal domains:

- General requisites: needed for impartiality and confidentiality of biobank operations;
- Structural prerequisites: governance structure, roles, and QMS;
- Resource essentials: personnel, facilities, equipment, and training;
- Process demands: costs for biospecimens and data handling, coupled with data protection policies;
- QMS: internal audits, risk assessment, planning, execution, and continuous monitoring.

It should be noted that simple adherence to ISO 9001 is not sufficient to demonstrate a biobank's competence in generating technically reliable data and outputs, as clarified in Appendix C of ISO 20387 [19]. ISO 15189:2022 transcends conventional risk management by incorporating the identification and utilization of opportunities, thereby advancing operational efficiency and patient care in medical laboratories. Section 5.6 mandates that laboratories establish procedures for managing both risks and opportunities, fostering proactive improvements. This dual emphasis empowers laboratories to refine their management frameworks through innovative practices, enhanced quality control measures, and streamlined workflows. The standard cultivates a culture of continuous improvement, leading to sustained performance enhancements and better patient outcomes. By integrating risk and opportunity management, ISO 15189:2022 ensures that laboratories are protected against potential threats while continuously evolving to meet new challenges, ultimately benefiting both patients and personnel. The efficiency of biobank centers on a comprehensive QMS embracing quality assurance and quality control programs across its spectrum of activities [20]. Crucial to this system are foundational principles and regulations that underpin the QMS, including a process-oriented approach, continual QMS enhancements, supervision of processes, allocation of essential resources, and stakeholder-centric focus [19, 42, 43]. To ensure QMS effectiveness, the biobank should institute comprehensive standard operating procedures covering core and ancillary processes, instrumental in providing operational efficiency and QMS efficacy [32].

### 2.5 Data Protection for Ethical Biobanking

The QMS alignment with GDPR (or its equivalent in non-European settings) and the constellation of local regulations, best practices, recommendations, guidelines, and directives from data protection authorities is extremely challenging but required for ethical biobanking [44-46]. The DPO can leverage the quality system to conduct audits, verify compliance with procedures, identify potential privacy-related nonconformities, assess the effectiveness of security measures, and report findings [44, 45]. This information can aid in planning corrective actions, according to the ISO 20387 [19, 20]. Joint actions and additional measures are necessary to ensure an integrated management system and an appropriate level of security in line with the associated risks. One of the most important changes introduced in the latest version of ISO 9001:2015 is the introduction of a systematic approach to managing risk rather than treating prevention as a separate component of the QMS. Data controllers should conduct a Data Protection Impact Assessment (DPIA), implement measures to mitigate the risks identified and consult with data protection authorities where such DPIAs identify high risks that cannot be softened [47]. Controllers and processors may also be responsible for the joint appointment of a DPO. This obligation applies to biobanks and biobank researchers if their core activities involve the processing of personal data. It also ensures greater consistency in assessments and preventive measures by ensuring that all parties involved have common procedures and a shared understanding of the risks, as well as a continuous effort to improve risk management and data protection while reducing the risk of non-compliance. In Europe, conducting a deep and detailed DPIA, mandated by GDPR Articles 35 and 36, is essential for ethical biobanking [48]. When selecting a Laboratory Information Management System (LIMS) for sample data, it is recommended to follow local guidelines, if available (*e.g*., Italian Guarantor Authority Provision No. 146 (2019) on genetic data. Finally, the coordination between the Data Protection Officer (DPO), data protection expert, and data controller in biobanks cannot be overstated. They are the guardians of compliance with data protection regulations, the protectors of individual privacy, and the stewards of data security. Their expertise ensures that sensitive biological and health-related data are collected, used, and shared for research purposes in an ethical and responsible manner. Collaboration among these professionals is vital for maintaining stakeholder trust, safeguarding research integrity, and propelling biomedical science forward while upholding ethical standards. In the intricate landscape of biobanking, their roles are indispensable pillars supporting the foundation of ethical data management and advancing the frontiers of scientific discovery.

### 2.6 Transparency and Consent Framework

To ensure alignment with ISO 20387 and pertinent privacy regulations, it is recommended to maintain transparency when summarizing the complete activity and data processing cycle in the information document furnished to potential donors [49]. This document consists of two distinct parts, as specified by the European Data Protection Board (https://edpb.europa.eu/) in Opinion 3/2019. In both scenarios, it is essential to establish a clear demarcation between research data or genetic data on one hand and sensitive research data on the other [50]. Additionally, it is imperative to provide an account of the research conducted within the home institution [51]. Under European data protection law, there is a common misconception that processing personal data for scientific research always requires explicit consent. Article 9(2)(j) of the GDPR provides an exemption for the processing of special categories of data, such as health-related information, in scientific research.52 On the other hand, the regulation lacks explicit guidance on compliance, leading to ambiguity and heterogeneity in its interpretation and subsequent application [52, 53]. Despite providing multiple legal bases in Article 6, of which consent is only one aspect, the GDPR emphasizes the need for enhanced protection for certain categories of data, such as health-related information, which requires compliance with Article 9(2) exemptions [52]. The 'research exemption' under Article 9(2)(j) allows for the processing of special categories of data for scientific research, but its vagueness regarding compliance measures creates uncertainty among legal requirements [52, 53]. Codes of conduct play a crucial role in harmonizing GDPR standards with national laws governing biobanking and scientific research to maintain standardized principles across jurisdictions [54]. They define the exceptions allowed by national laws and highlight instances where legislation may impede scientific purposes, thus ensuring coherence and consistency of regulations [54]. Advocates for patient rights have criticized the use of tissues in various research, whether for scientific or for-profit purposes, highlighting concerns about participant autonomy and property rights over the sourced tissue [55]. There is an ongoing debate regarding the necessity of explicit consent for such samples. However, it's worth noting that while informed consent is generally preferred, ethical oversight committees can waive this requirement if they ascertain minimal risks to subjects and find re-consent impractical [55].

#### 2.6.1 GDPR Consent for Biobanking Research and/or Experimentation

This part serves as an ethical standard and procedural obligation, describing the activity and its potential benefits. It may also include information about compliance with ISO 20387 or ISO 9001 certification to inform prospective donors about the biobank's commitment to quality. However, this consent does not cover the processing of personal data. According to Articles 5, 12, and 13 of the GDPR, the data controller should fulfill their transparency obligation by providing all the necessary information specified in Article 13 of GDPR. Consent to data processing, as the lawful basis for processing, requires a separate document. In addition, the GDPR provides robust data protection and privacy rights, including genetic data behind genetic discrimination. In the United States, the Genetic Information Nondiscrimination Act (GINA) of 2008 prevents genetic discrimination in health insurance and employment, promoting genetic testing and research participation without fear of discrimination.

Other countries have also legislation addressing genetic discrimination. Canada's Genetic Non-Discrimination Act (2017) is similar to GINA, prohibiting the use of genetic information in insurance and employment. Australia has guidelines preventing health insurers from requiring genetic tests but allows the use of existing genetic information under certain conditions. Japan lacks comprehensive legislation but has guidelines discouraging genetic discrimination. Overall, these legal frameworks play a crucial role in fostering trust and ensuring that the benefits of genetic research are accessible and applicable to diverse populations worldwide, ultimately improving health outcomes on a global scale.

#### 2.6.2 GDPR Consent for Data Processing

Drafting this document is challenging, as it requires summarizing how the data will be used, the purposes and methods of processing, the entities involved, the security measures (including pseudonymization), and the process for revoking consent. The document also covers communications to third parties and international transfers, including the modalities and safeguards involved. The difficulty lies in communicating this information in a clear and easily understandable manner, ensuring that the document is not merely a formal requirement. It should effectively convey the care taken by the data controller when processing sensitive data. Aligning compliance efforts with ISO 20387 and GDPR is the recommended approach to avoid duplicative and inconsistent work in terms of procedures and identifying appropriate security measures.

## 3 INTELLECTUAL PROPERTY

Differing viewpoints exist regarding the extent of openness or restriction of intellectual property in biobanking. With a primary focus on upholding individuals' privacy, biobanks function as collaborative systems among academic research groups, the public sector, individual participants (not limited to patients), and industry. They facilitate the sharing of both information and samples, creating a valuable resource for the scientific and medical community, ultimately benefiting society as a whole. Fig. (**1**) outlines the key steps in the biobanking process, starting with the written consent, followed by sample collection, processing, and the generation of new intellectual property [56-58]. Regrettably, the legal system has yet to provide a satisfactory resolution to this ongoing debate. Researchers can request biological materials for their scientific endeavors, always prioritizing the utmost protection of individuals from whom the biological materials were obtained and belong, as long as their usage remains within the boundaries of consent. Particular attention should be given when utilizing biological material that may lead to inventions eligible for patents and potential commercialization [59]. Ownership of such inventions is normally determined according to the inventive contribution of all parties involved in their generation. No ownership or economic benefit is shared with the donors. Such inventions can result in one or more commercial products and represent a valuable commercial asset for a research institute. They attract the interest of industrial partners who invest in further development through the “patent bargain,” a cornerstone of the patent system. In essence, society grants exclusivity to inventors in exchange for knowledge sharing, promoting ongoing innovation and societal progress. Yet, advancements in biology, especially genetics, have introduced complex questions regarding the patentability of living matter and biological materials. European Directive 98/44/EC addresses these issues, establishing that certain inventions involving the human body and its genome are non-patentable to protect human dignity and genetic integrity (article 6, 98/44/EC). However, elements isolated from the human body or created through technical processes may be patentable if their industrial application is described in the patent application (article 5, 98/44/EC). Importantly, any patent application concerning biological material of human origin should be accompanied by the express, free, and informed consent of the individual from whom the material was obtained in accordance with the national law. The objective of securing patent rights should always align with the fundamental principles of respecting human dignity and integrity.

### 3.1 Enhancing Biobank Research through Strategic Collaborations: Opportunities and Challenges

The biobanks are indispensable tools in modern biomedical research. They provide the infrastructure necessary for conducting high-quality, large-scale studies that drive scientific discovery and innovation. Through their role in facilitating collaboration, supporting longitudinal and population health studies, and ensuring ethical research practices, biobanks significantly contribute to our understanding of health and disease, ultimately improving patient outcomes and public health.

The utilization of biobank samples has significantly contributed to numerous research articles, aiding in uncovering genetic factors underlying diseases, advancing personalized medicine, and identifying new therapeutic targets [1]. This data has enabled groundbreaking discoveries and innovations in medical research, enhancing our understanding of disease mechanisms and paving the way for effective treatments. For instance, our biobank played a crucial role during COVID-19 by developing a test to identify immunoglobulins in patients, as detailed in a study published [2]. This test has become a critical tool in diagnosing and monitoring the immune response to COVID-19, highlighting the invaluable role of biobank resources in addressing urgent public health challenges [2].

In Italy, biobanks have advanced research in rare genetic disorders, neurodegenerative diseases, cancer, and cardiovascular conditions. The Italian Network of Genetic Biobanks (TNGB) has identified mutations for disorders like Duchenne muscular dystrophy and cystic fibrosis [3]. The Sapienza University of Rome Biobank has been influential in cancer and cardiovascular research [4]. At the same time, the San Raffaele Telethon Institute for Gene Therapy (TIGET) Biobank has advanced gene therapy and immunotherapy research [5]. Our biobank plays a vital role in research at our institute and in collaboration with various research centers and companies. Through these collaborations, numerous publications have emerged, significantly advancing our understanding of various health conditions.

Internationally, the UK Biobank has provided insights into obesity and severe COVID-19 outcomes [6]. The Estonian Genome Project has contributed to personalized medicine and revealed risk factors for various diseases [7]. Icelandic deCODE Genetics discovered a mutation protecting against Alzheimer’s and identified variants linked to cardiovascular diseases [8]. Finnish Biobanks, through the FinnGen project, have uncovered variants for rare neuromuscular disorders and enhanced understanding of the Finnish population's genetic structure [9]. BioBank Japan has identified genetic variants associated with cancers and chronic diseases, aiding in tailored prevention and treatment strategies [10].

Collaboration between countries across the globe is paramount to advancing genomic research and improving healthcare outcomes. Particularly, partnerships between European countries and ASEAN countries, which are often considered second-tier in genomic research, can yield significant mutual benefits. These collaborations are not only vital for sharing advanced technology, expertise, and funding but also for fostering a comprehensive understanding of genetic diversity. Such international cooperation is essential for the development of inclusive and effective personalized medicine. Furthermore, collaborations with Asian countries are especially important due to their diverse populations and unique genetic profiles, which can provide critical insights into genetic variations and disease mechanisms. The following text delves into the multifaceted benefits of this collaboration, emphasizing the crucial role of diverse genetic data and shared resources in accelerating scientific discoveries and enhancing global healthcare.

Collaboration between European countries and ASEAN countries in genomic research offers numerous mutual benefits. Firstly, European countries can provide advanced technology, expertise, and funding, which can significantly boost the research capabilities of ASEAN countries. Joint research projects and consortiums enable scientists from both regions to work together on critical health issues, sharing data and resources to accelerate discoveries.

One of the primary benefits of such collaboration is the enhancement of personalized medicine through the inclusion of diverse genetic data. Personalized medicine aims to tailor treatments to individual genetic profiles, but its effectiveness is limited if the research is based predominantly on data from one population. By gathering genomic data from multiple countries and ethnicities, researchers can identify a wider range of genetic variations and their associations with diseases. This inclusivity ensures that personalized treatments are effective across different populations, including minorities, who are often underrepresented in research.

For example, minority populations in the global north may have unique genetic markers that influence their response to certain medications. Without adequate research, these groups may not benefit from the advances in personalized medicine. Collaborating with ASEAN countries, which have diverse populations, allows for a more comprehensive understanding of genetic variations, leading to more effective and inclusive healthcare solutions.

Additionally, such collaboration fosters global scientific advancement by pooling knowledge and resources, leading to faster innovations and improved healthcare outcomes. This not only benefits the participating countries but also contributes to the global effort in combating genetic diseases and improving public health.

In conclusion, the collaboration between European and ASEAN countries in research is crucial for advancing personalized medicine. By including diverse populations, we can ensure that medical treatments are effective for everyone, thereby reducing health disparities and enhancing the overall quality of healthcare globally.

## CONCLUSION

In conclusion, biobanks serve a pivotal role in advancing scientific research, providing that they are structured to safeguard individuals' privacy rights. Striking a balance between scientific exploration, the right to health, personal autonomy, and privacy is a multifaceted endeavor requiring careful legal and ethical deliberation. The existing legal framework, though not without flaws, provides crucial guidelines for the establishment and operation of biobanks, highlighting the importance of transparency, donor consent, and adherence to privacy regulations. Moreover, regulatory initiatives like ISO 20387:2018 accreditation have significantly influenced the biobanking landscape. This accreditation, by standardizing procedures, fortifying QMS, and fostering global collaboration for research integrity, has notably enhanced the ethical and operational standards within the field. However, it is important to note that this framework is not always uniform across various regions of the world and, regrettably, is not universally implemented.

For this reason, there remains ample space for further development and refinement, particularly concerning the patentability of biological materials and the ethical intricacies surrounding human bodies. To ensure the continued moral and responsible growth of biobanks, interdisciplinary collaborations, meaningful engagement with stakeholders, and updates to legal frameworks are imperative. Given the multitude of facets, we've examined and recognized the fundamental role of the individual participants in the broader biobanking context of the modern era. This approach not only addresses individual health needs but also contributes to the betterment of society as a whole.

## AUTHORS’ CONTRIBUTIONS

AC drafted the manuscript. G.B., E.S., A.A., L.O.M., F.P., M.F., C.R.M., C.F., O.P., E.G.R., M.M., O.R., and N.F. contributed to iconography. N.F. supervised the study. All authors have approved the submitted version of the manuscript.

## Figures and Tables

**Fig. (1):**
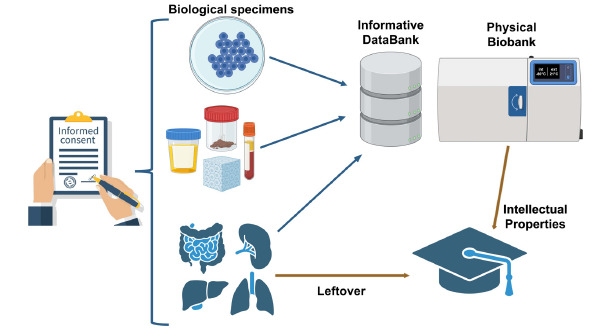
Major steps in biobank process which includes the acquisition of consent, sample collection, processing, and finally generation of new intellectual property. **Abbreviation**: LIMS, Laboratory Information Management System.

## LIST OF ABBREVIATIONS

DPIA: Data Protection Impact Assessment
DPO: Data Protection Officer
EU: European Union
FFPE: Formalin-fixed Paraffin-embedded
GDPR: General Data Protection Regulation
GINA: Genetic Information Nondiscrimination Act
HIPAA: Health Insurance Portability and Accountability Act
ICH: International Council for Harmonization
ISBER: International Society for Biological and Environmental Repositories
LIMS: Laboratory Information Management System
QC: Quality Control
QMS: Quality Management System
TIGET: Telethon Institute for Gene Therapy
TNGB: The Italian Network of Genetic Biobanks
WHO: World Health Organization
